# Glioblastoma Multiforme: Probing Solutions to Systemic Toxicity towards High-Dose Chemotherapy and Inflammatory Influence in Resistance against Temozolomide

**DOI:** 10.3390/pharmaceutics15020687

**Published:** 2023-02-17

**Authors:** Sadia Nasir, Sadia Nazir, Rumeza Hanif, Aneela Javed

**Affiliations:** Department of Healthcare Biotechnology, Atta-ur-Rahman School of Applied Biosciences (ASAB), National University of Sciences and Technology (NUST), H-12 Campus, Islamabad 44000, Pakistan

**Keywords:** glioblastoma, chemotherapy resistance, solid lipid nanoparticles, inflammatory markers, drug delivery, temozolomide

## Abstract

Temozolomide (TMZ), the first-line chemotherapeutic drug against glioblastoma multiforme (GBM), often fails to provide the desired clinical outcomes due to inflammation-induced resistance amid inefficient drug delivery across the blood-brain barrier (BBB). The current study utilized solid lipid nanoparticles (SLNPs) for targeted delivery of TMZ against GBM. After successful formulation and characterization of SLNPs and conjugation with TMZ (SLNP-TMZ), their in-vitro anti-cancer efficacy and effect on the migratory potential of cancer cells were evaluated using temozolomide-sensitive (U87-S) as well as TMZ-resistant (U87-R) glioma cell lines. Elevated cytotoxicity and reduction in cell migration in both cell lines were observed with SLNP-TMZ as compared to the free drug (*p* < 0.05). Similar results were obtained in-vivo using an orthotopic xenograft mouse model (XM-S and XM-R), where a reduction in tumor size was observed with SLNP-TMZ treatment compared to TMZ. Concomitantly, higher concentrations of the drug were found in brain tissue resections of mice treated with SLNP-TMZ as compared to other vital organs than mice treated with free TMZ. Expression of inflammatory markers (Interleukin-1β, Interleukin-6 and Tumor Necrosis factor-α) in a resistant cell line (U87-R) and its respective mouse model (XM-R) were also found to be significantly elevated as compared to the sensitive U87-S cell line and its respective mouse model (XM-S). Thus, the in-vitro and in-vivo results of the study strongly support the potential application of SLNP-TMZ for TMZ-sensitive and resistant GBM therapy, indicatively through inflammatory mechanisms, and thus merit further detailed insights

## 1. Introduction

Gliomas are the most lethal, incurable, and malignant primary brain tumors of glial cells. Malevolent deregulation of the Glia leads to tumor development [1]. Among diffuse gliomas, Glioblastoma multiforme (GBM), a parenchymal tumor, is the most recurring and deadliest malignancy, with a poor prognosis. Nearly 54% of gliomas are GBM [2]. The invasive nature of GBM restricts its surgical resection, leaving tumors behind in the complicated parts of the brain. Therefore, concomitant radiotherapy and chemotherapy are practiced following surgery. Problems with chemo- and radiotherapy arise with the acquisition of inherent and acquired resistance in tumor cells [3].

Spontaneous conversion of a pro-drug to an active form in the blood reduces its long-term activity [4], which limits its benefits; other reasons are the ability to cross the blood-brain barrier (BBB) efficiently [5], higher efflux potential than an influx of the drug in tumor cells [6], a deregulated apoptosis mechanism [7], and dose-limiting effects, caused by repeated and high-dose exposure to tumor cells for maintaining its cytotoxic action. Also, numerous side effects have been reported, including myelosuppression, nausea, and cognitive decline [8]. To reduce these side effects and to increase the active time span of a drug, drug delivery carriers are proposed to be used in this study.

Drug delivery nano-systems have been gaining massive attention for the treatment of cancer, especially in GBM, as they have been observed to be efficient in drug delivery compared to conventional formulations currently available in the market [9].

Lipid-based nanoparticles comprising physiological lipids are being used as drug delivery vehicles into the brain due to their biodegradable and biocompatible nature [10]. Among these, solid lipid nanoparticles (SLNPs) carry high drug delivery potential. SLNPs are lipid-based oily droplets stabilized by surfactant [11] and can easily fuse to endothelial cells to cross the BBB in the brain. The advantages are; it carries a high drug load, carries insoluble drugs [12], it is more stable with no drug leakage, it has a timely release of the drug, has no toxicity, it is cost-effective [13], and is convenient for preparation and administration [12].

Temozolomide (TMZ), an imidazotetrazine, is a pro-drug that acts as an alkylating agent; it is used as the first line of defense in GBM [14]. The treatment benefits of TMZ are limited due to acquired resistance, a process that remains incompletely understood [15]. The drug resistance phenomenon is an immense obstacle to GBM management. The cytotoxic effect produced by TMZ in GBM is also hampered by DNA repair mechanisms. Defects in the mismatch repair (MMR) pathway allow cell survival and proliferation rather than initiating cell death/apoptosis in response to TMZ [16]. This study discusses the systemic toxicity of TMZ when administered alone and with lipid-based nanocarriers. The effectivity of SLNPs in the case of TMZ-sensitive and TMZ-resistant GBM tumor cells is evaluated. In the literature, solid lipid nanoparticles have been used previously for conjugating various chemotherapeutics, but little has been studied on the effect of SLNP-conjugated TMZ in resistant GBM cells.

The second objective of the current study is to shed light on the micro-environment of tumors that consists of inflammatory chemokines and cytokines, and plays a powerful role in the initiation and progression of cancer [17]. Inflammation is the seventh hallmark of cancer, which leads to a proposal of its connection with acquired chemo-resistance in GBM [18]. GBM is circumscribed by growth factors, chemokines, and pro-inflammatory cytokines. The role of inflammation has been reported in developing chemo-resistance [19]. TMZ treatment has also been found to increase the expression of pro-inflammatory cytokines with an oncogenic role. Long-term treatment with TMZ has been shown to develop resistance in astroglia by the activation of inflammatory chemokines. Hence TMZ-induced production of pro-inflammatory cytokines such as interleukins (IL) IL-1β, IL-6 and Tumor necrosis factor-α (TNF-α) may also be suspected as a cause of drug resistance [20].

One of the two objectives of this study was to analyze the efficiency of solid lipid nanoparticles (SLNPs) in delivering a chemotherapeutic drug into the brain and analyze its systemic toxicity. The other was to highlight the levels of inflammatory markers that are potentially one of the reasons for the chemo-resistant nature of tumors.

## 2. Materials and Methods

### 2.1. Chemicals

Soya lecithin, stearic acid, polysorbate-80 (PS-80), isopropanol, phosphate buffer saline (PBS-P4417), Chloroform, Methanol, Temozolomide (TMZ) were from Sigma-Aldrich (St. Louis, MO, USA); Dulbecco’s modified Eagle’s medium (DMEM) was obtained from GIBCO (Grand Island, NY, USA). Fetal bovine serum (FBS) and 1% penicillin/streptomycin were purchased from Invitrogen (Thermo Fisher Scientific, Inc., Waltham, MA, USA); 0.22 µm syringe filter (Millex-GS Millipore). All other chemicals used were of analytical grade.

### 2.2. Development of TMZ Resistant U87 (U87-R) Cell Line

The human-derived U87-MG cell line cells were from the American Type Culture Collection (ATCC; Manassas, VA, USA). Cell lines were cultured in DMEM with 10% FBS, and 1% penicillin/streptomycin (100 units/mL), at 37 °C in a 5% CO_2_-humidified atmosphere. The temozolomide (TMZ)-resistant cell line (U87R-MG) was developed by treating the parental cell line (U87-MG) with increasing concentrations of TMZ (up to 960 µM) for 72 h. Periodic washout was followed with a drug-free medium for 72 h. The procedure was repeated for 8 weeks, ensuring that only the resistant cells survived and outgrew the cultures.

### 2.3. Preparation of Solid Lipid Nanoparticles (SLNPs)

**Figure 1:**
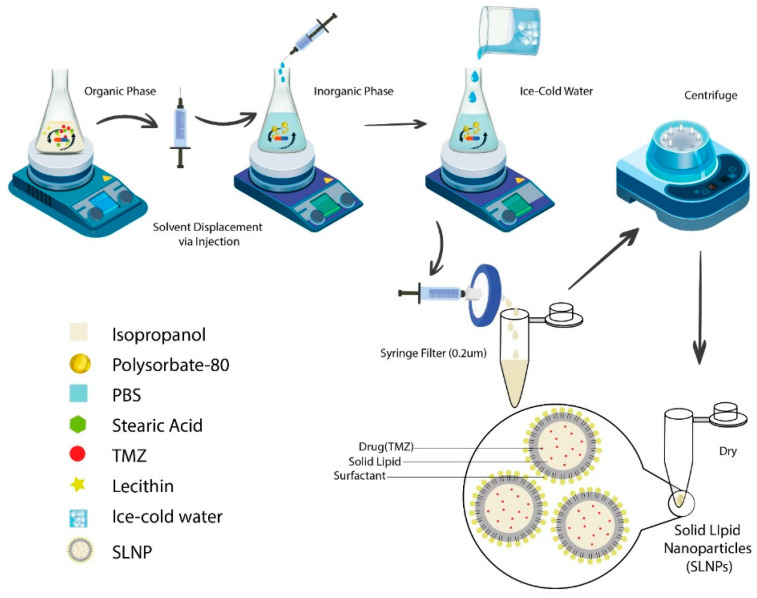
Graphical representation of the formation of temozolomide-loaded solid lipid nanoparticles (SLNP-TMZ) via the hot injection method and emulsification protocol. The ingredients of the organic and inorganic solutions were mixed separately using a hot magnetic plate. The organic phase was dispersed into an inorganic phase drop-by-drop via injection. Ice cold water was added, the solution was centrifuged, and the pallet was dried and stored.

The hot solvent injection method was used for the preparation of SLNPs [21]. Briefly, SLNPs were made by mixing the organic phase (stearic acid (100 mg), lecithin (150 mg), temozolomide (24 mg)) in isopropanol (20 mL) and an inorganic phase (1% Polysorbate-80 solution in PBS (100 mL)) via injection (24 gauge) on a magnetic stirrer at 700 rpm, stirring continuously for an hour. Ice cold water (5 mL) was added, and the solution was filtered through a 0.2 um filter. The filtrate was centrifuged at 20,000 rpm at 4 °C for one hour, and the obtained pallet was dried and stored at −20 °C (Figure 1). For the preparation of blank SLNPs, the same procedure was followed, excluding the drug. SLNPs were prepared in a horizontal laminar flow hood under sterile conditions to perform the in-vitro cell line studies [22].

### 2.4. Characterizing SLNPs by Physio-Chemical Techniques

SLNPs and SLNP-TMZ were characterized scanning electron microscopy (SEM) and elemental diffraction spectroscopy (EDS) (for defining the size and elemental status of the SLNPs, respectively). Functional groups were assessed by Fourier transform infrared spectroscopy (FTIR), and the crystallinity of the SLNPs was analyzed by X-ray Diffraction Analysis (XRD).

#### 2.4.1. Encapsulation Efficiency (EE) and Drug Release (DR) of SLNP

Entrapped temozolomide in the SLNP’s formulation was centrifuged and filtered through a 0.2 µm syringe filter. The calibration curve for temozolomide was established by dissolving different amounts of TMZ in a DMSO solution, and the absorbance was noted for each sample by using a UV-Vis spectrophotometer according to Beer’s law, and a standard curve was drawn. The absorbance and amount of TMZ were plotted on a graph, and the unknown values of absorbance for entrapment efficiency were calculated from the calibration curve. The un-entrapped drug was determined by a UV-Visible spectrophotometer at λ330 nm wavelength. The entrapped drug was calculated by using the formula given below;

EE (%) = D_(Un-entrapped)_ × 100/D_total_

where, D_(Un-entrapped)_ = drug in the supernatant and D_total_ = total drug added in the SLNPs.

Different amounts of SLNPs were weighed (1 mg, 5 mg, and 10 mg), dissolved in 1 mL DMSO, incubated at room temperature for an hour in a shaking water bath, and centrifuged for 30 s (mini spin). The absorbance of the supernatant was checked, and the drug release was calculated according to Beer’s law principle, as in [22]:

Y = mx + c

#### 2.4.2. Cytotoxicity Assay to Determine TMZ Resistance

A 3-(4,5-dimethylthiazol-2-yl)-2,5-diphenyl-2H-tetrazolium bromide (MTT) colorimetric assay was used for determining cell viability. The cell viability of the U87-S and U87-R was checked in the presence of TMZ at different concentrations. Both cell lines were plated separately in a 96-well plate at a density of 1 × 106 cells/well in a prepared DMEM medium, and cells were allowed to adhere overnight. The medium was removed, and fresh TMZ-containing medium at different concentrations (0.01–1000 µM) was dispensed in wells. After 24 h, MTT reagent was added (5 mg/mL), and the medium was subjected to a 4 h incubation in the dark at 37 °C. The medium was then discarded, and DMSO was added to dissolve violet formazan crystals for 15 min. Absorbance was measured at 590 nm via a microplate reader (BioRad). Each experiment was done in three replicates. The viability percentage was calculated using the following equation,

% Viability = (OD treated well [−blank])/(mean OD control well [−blank]) × 100

### 2.5. Cell Migration by Wound Healing Assay

Sensitive and resistant cells were cultured in six-well plates with a density of 1 × 10^6^ cells/well. When cells grew to about 90% per well, 10 μL pipette tips were used to scratch the monolayer of cells. After washing cell debris with PBS, cells were cultured in 2% FBS medium in the absence or presence of the various concentrations in each of the following groups for 24 h: (1) Control; (2) TMZ; (3) SLNP-TMZ. The cells were photographed using a light microscope and a digital camera.

### 2.6. Animal Studies

Male Balb/c mice (8 weeks old) were purchased from the National Institute of Health (Islamabad, Pakistan) and were kept in the animal house facility of ASAB (NUST), Islamabad. The temperature was maintained between 22 °C and 28 °C with a 12/12 h light and dark period. Animals were fed ad libitum with commercially available food, and six mice were kept per group for the study. All procedures were reviewed and approved by the Institutional Review Board (IRB) Atta-ur-Rahman School of Applied Biosciences (ASAB), National University of Sciences and Technology (NUST), Pakistan, according to the verdict of the Institute of Laboratory Animal Research, Division on Earth and Life Sciences, National Institute of Health, USA (Guide for the Care and Use of Laboratory Animals: Eighth Edition, 2011).

#### 2.6.1. Pharmacokinetic Analysis (Tissue Distribution)

Equal amounts of TMZ in PBS and SLNP-TMZ were administered to normal Balb/c mice intraperitoneally at different time points (0, 2, 4, and 6 h). After euthanasia, a necropsy was carried out; the mouse heart, liver, spleen, kidneys, and brain were collected, snap-frozen, and stored at −20 °C. Each organ was minced separately in a mortar and pestle and added to an organic solution of methanol and chloroform (2:1 *v*/*v*). Tubes were incubated at 37 °C for 15–20 min in an orbital shaker for agitation. Afterwards, they were centrifuged for 10 min at 2000 rpm at 4 °C. An organic phase that contained the drug was collected and washed with 0.9% NaCl solution, and the optical density of each one was taken at 330 nm (the wavelength for temozolomide) using a UV-Vis spectrophotometer to determine the concentration of the drug in each organ.

#### 2.6.2. Generation of an Orthotopic Xenograft Mouse Model

A modified procedure from [23] was used to generate an orthotopic xenograft mouse model. U87 cells (TMZ-resistant) and sensitive cells were grown in DMEM containing 2.5% fetal calf serum, the medium was removed, and the cells were rinsed with PBS. They were then trypsinized, pelleted, and resuspended in PBS at 10^5^ cells/µL. They were kept on ice until they were injected intracranially into mice with a Hamilton syringe (Hamilton Company, Reno, Nevada).

**Figure 2:**
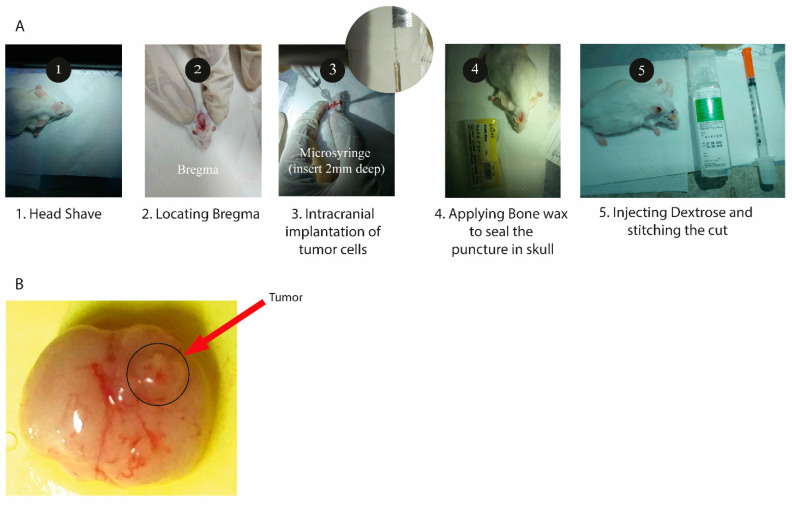
(**A**) Steps for the development of glioblastoma multiforme mouse model. (**B**) Mouse brain with a tumor developed in the right cerebrum (dorsal view).

**Figure 3:**
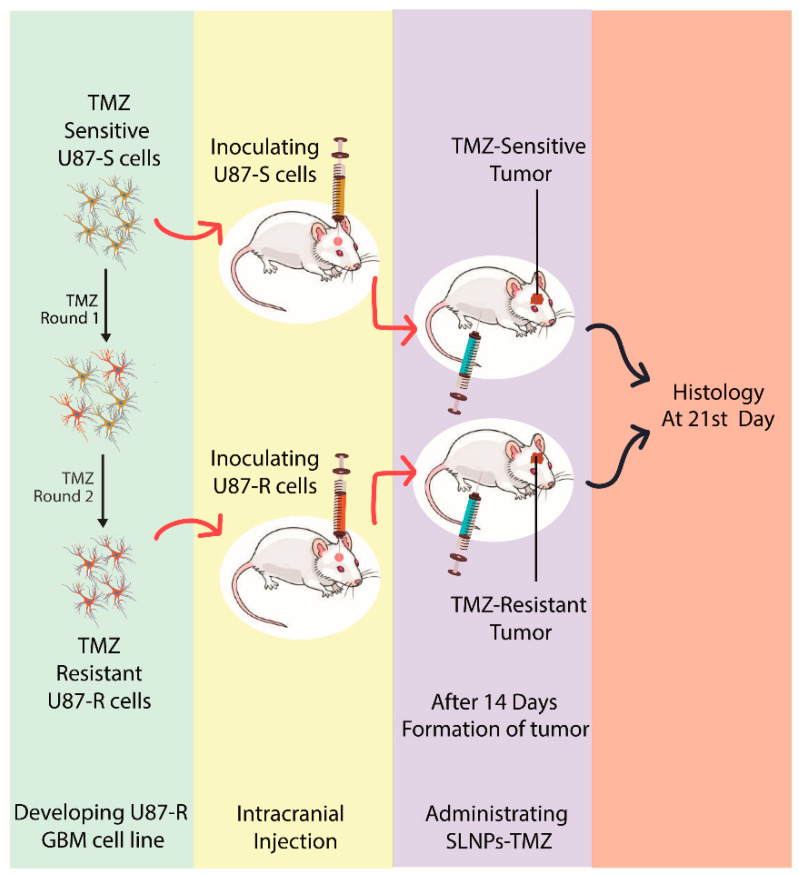
Graphical representation of intracranial inoculation of glioma using U87-S and U87-R and its treatment with SLNPs-TMZ and free TMZ.

The mouse was anesthetized, and its calvarium was exposed through a midline incision, and a burr hole was drilled 1 mm laterally (right) and 2 mm anteriorly to the bregma. Cells were injected at a rate of 1 µL/min (total of 10^8^ cells injected per mouse), with the syringe left in place for 2 to 3 min following the completion of the tumor cell injection. Injections were to a depth of 2 mm below the outer table of the skull, thereby introducing the tumor cells near the right caudate nucleus (Figure 2). Following tumor cell injection, mice were observed daily until they reached a moribund state, at which time they were euthanized, and their brains removed and processed for histopathologic analysis (Figure 3).

#### 2.6.3. Drug Treatment Regimens and Doses

After 7 days of cell implantation, SLNP-TMZ and free TMZ were administered to the mice (six mice in each group) with sensitive and resistant tumors intraperitoneally, TMZ; 60 mg/kg 5 days a week and SLNP-TMZ (dose calculated from drug load in SLNPs, equal to the plain TMZ) [24] as stated in Table 1.

#### 2.6.4. Hematoxylin and Eosin Staining

Post-treatment (after 21 days) analysis of the tumor was carried out using staining techniques. The whole brain was harvested and stored in 4% paraformaldehyde solution (freshly prepared) for 24 h at 4 °C. Tissue dehydration and fixation were carried out using alcohol of different concentrations, and samples were paraffinized and sectioned (5 µm thick). Hematoxylin and Eosin staining was performed and evaluated using light microscopy.

#### 2.6.5. Differential Quantification (qRT-PCR) of Inflammatory Markers

Total RNA was isolated from U87-S and U87-R cell lines (invitro) and dissected tumors from in vivo mice models (after 7 days of inoculation), by TRIzol Reagent (Invitrogen). RNA (1 µg) was retrotranscribed using the using Revert-Aid^TM^ qPCR kit (Thermo Scientific^TM^, Waltham, MA, USA). IL-6, IL-1β, and TNF-α expression were evaluated with a gene expression assay to detect a change in the expression of inflammatory cytokines in resistant cell lines and tumors derived from resistant cell lines. MGMT and STAT3 were taken as resistance markers, and bcl-2 and Ki-67 were anti-apoptotic and proliferation markers, respectively. The β-Actin gene was used to normalize the cDNA amounts. Real-time PCR was performed using the ABI 7500 sequence detection system (Applied Biosystems) in triplicate for each sample in a 15 µL final volume containing 0.1 uL of diluted cDNA, 0.4 µL of TaqMan Universal PCR Master Mix and 1 µL primer each (Table 2) and 4 uL of reaction buffer. The results were analyzed with a ∆∆ threshold (ΔΔC_t_) cycle method (Livak method).

### 2.7. Statistical Analysis

Data are shown as mean ± SEM. Statistical analyses were performed with Prism 7.0 software (GraphPad Software, La Jolla, CA, USA) using one-way ANOVA and *t*-test. A *p*-value less than 0.05 was considered significant.

## 3. Results

### 3.1. Physiochemical Characterization of Solid Lipid Nanoparticles (SLNPs)

#### 3.1.1. Scanning Electron Microscopy (SEM)

**Figure 4:**
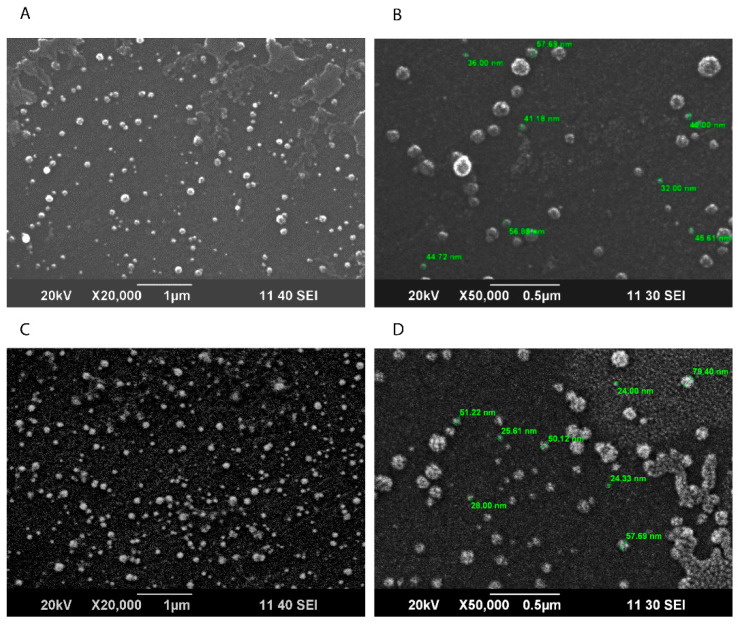
Scanning electron microscopy (SEM) micrographs, (**A**,**B**) TMZ-loaded SLNPs, at X20,000, scale bar 1 µm and X50,000, scale bar 0.5 µm and (**C**,**D**) Blank SLNPs at X25,000, scale bar 1 µm and X50,000, 0.5 µm.

The particle shape (roundness, smoothness, and formation of aggregates) of solid lipid nanoparticles was determined via SEM. Micrographs were taken at X50,000, and the spherical surface morphology was observed of varying sizes, with an average diameter of 55.20 nm (Figure 4).

#### 3.1.2. Fourier Transfer Infrared Spectroscopy (FTIR)

**Figure 5:**
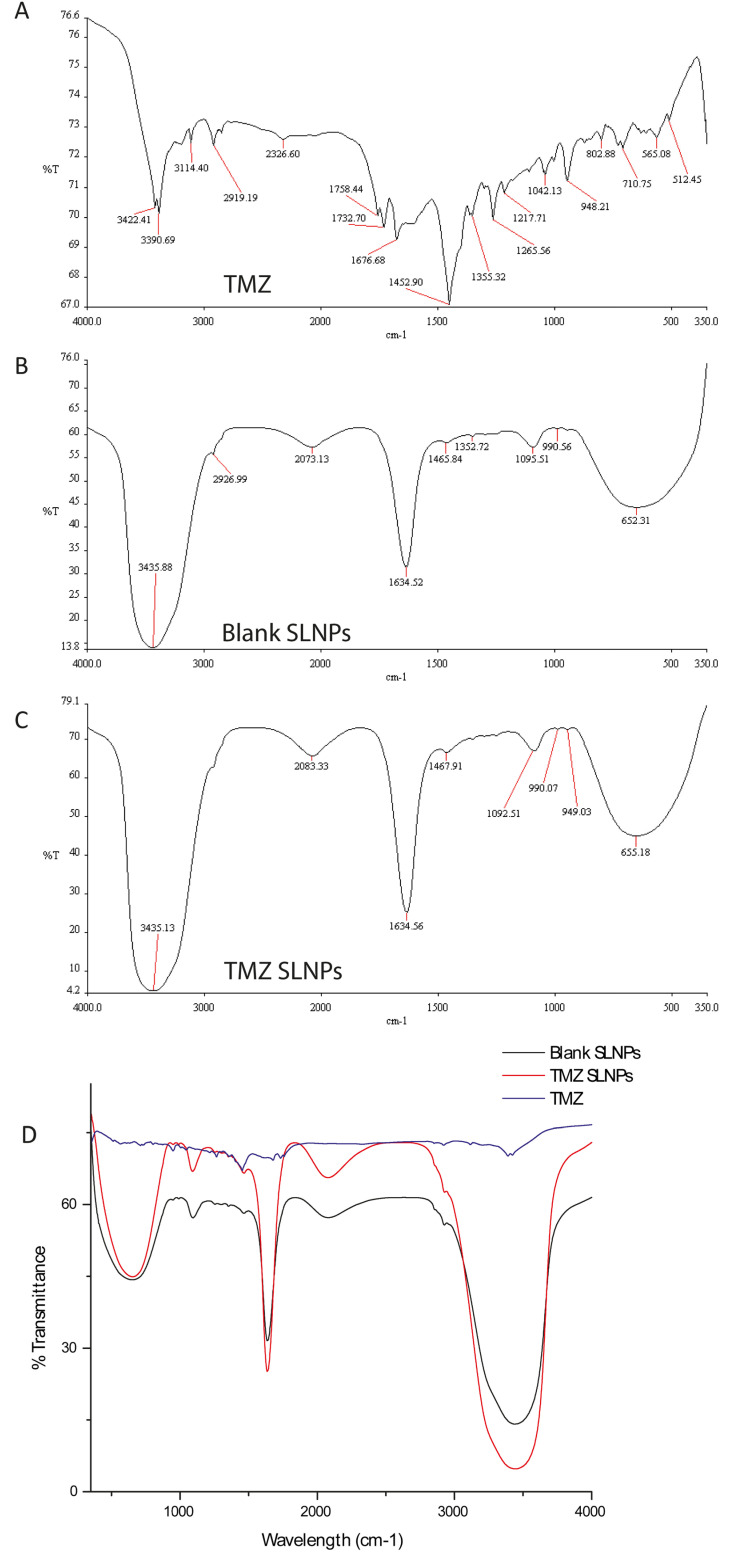
Fourier transform infrared spectroscopy (FTIR) spectra (**A**) TMZ. (**B**) Blank-SLNPs and (**C**) SLNP-TMZ, (**D**) Collective spectra of three plotted via Origin 8.0. The infrared spectra had strong absorption peaks, at 1600 cm^−1^ and 3500 cm^−1,^ that are C-C and N-H bonds in the case of SLNP-TMZ, which are stretching vibration peaks due to the conjugation of TMZ with SLNPs via coupling reaction.

FTIR was performed at wavelengths ranging from 4000–400 cm^−1^ to confirm the compatibility between the drug and the lipids. Characteristic peaks of TMZ lie at 3422 cm^−1^, 3390.29 cm^−1^ (this doublet shows the presence of the primary amide (N-H) group), 1452.90 cm^−1^ (-CH_3_), 1042 cm^−1^ (-C-CH_3_) and 948.21 cm^−1^ 710 cm^−1^ are due to the =C-H. Peaks in unloaded solid lipid nanoparticles represent N-H, O-H at 343.88 cm^−1^, C=C at 1634.52 cm^−1^, and 652.31 cm^−1^ representing -CH_2_. The same functional groups are present in both reactants of SLNPs, and FT-IR analysis of their product shows stretching of bands that give enhanced % transmittance at these wavelengths, which gives an idea of the coupling reaction between them (Figure 5).

#### 3.1.3. X-ray Dispersive Spectroscopy (XRDS)

**Figure 6:**
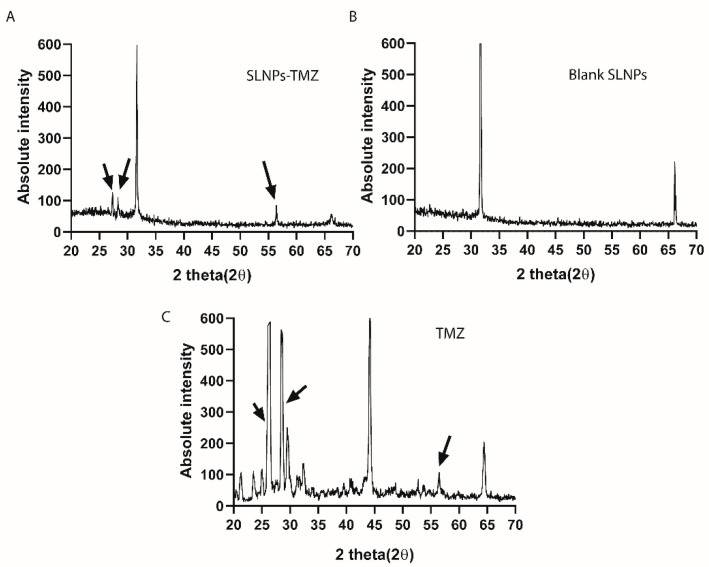
X-ray dispersive spectroscopy (XRDS), (**A**) SLNP-TMZ, (**B**) Blank SLNP, (**C**) X-ray Patterns of TMZ. The sharp peak between 26° and 28° of SLNP-TMZ suggests the amorphous nature of TMZ after conjugation with SLNPs. The arrows represent the changes in the peaks upon incorporation of TMZ into SLNPs.

Energy dispersive spectroscopy was performed to analyze the crystallinity of the drug after incorporation with SLNPs. The broadening peaks of the XRDS spectrograms are representative of the amorphous nature of the compound, whereas the sharp peaks are characteristic of crystallinity. The reduction in the SLNP-TMZ suggests the amorphization of TMZ when conjugated with SLNPs (Figure 6).

#### 3.1.4. Energy Dispersive Spectroscopy (EDS)

**Figure 7:**
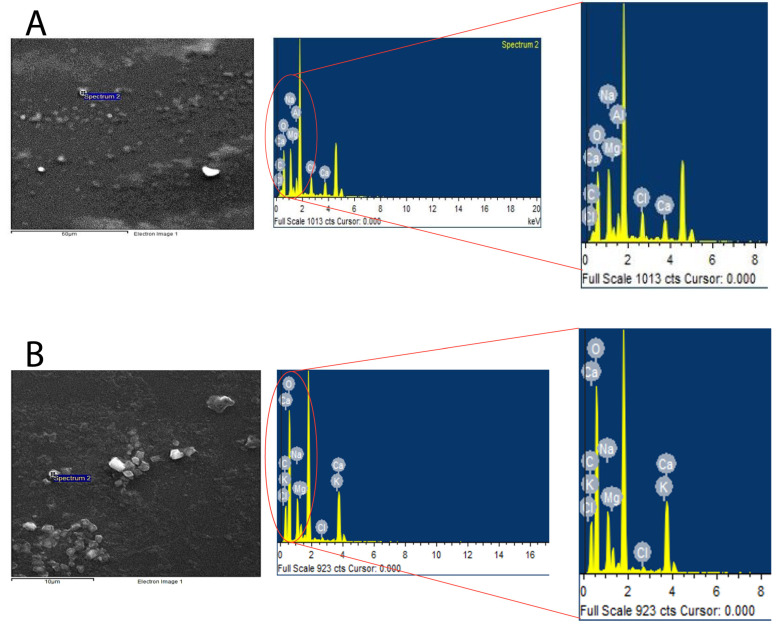
Energy dispersive spectroscopy (EDS) (**A**) Blank-SLNPs, (**B**) SLNP-TMZ. An increase in the peaks of Hydrogen and Oxygen could be observed in the spectrum of SLNP-TMZ, which could be explained by the incorporation of TMZ in the SLNPs.

EDS was performed to evaluate the elemental composition of the loaded and unloaded SLNPs, to deduce whether it was incorporated into nanoparticles or not, as the FT-IR indicated that they have common functional groups. Therefore, it was expected from performing EDS that a higher content of carbon and oxygen would be observed in loaded SLNPs compared to the unloaded ones (Figure 7 and Table 3).

#### 3.1.5. Entrapment Efficiency of SLNPs

Drug-loaded SLNPs were centrifuged, and the absorbance of the supernatant was taken and plotted on a pre-formed calibration curve to calculate the amount of entrapped drug;

EE% = 1 − (amount of drug in supernatants/amount of drug added) × 100

The EE% calculated was 94.47%, which shows that a significant percentage/amount of drug was entrapped in the SLNPs, and a little amount (5.53%) was observed in the supernatant, as calculated from the absorbance taken by UV-vis spectrophotometer at a specific wavelength of TMZ.

### 3.2. Drug Release Efficiency of SLNP-TMZ

**Figure 8:**
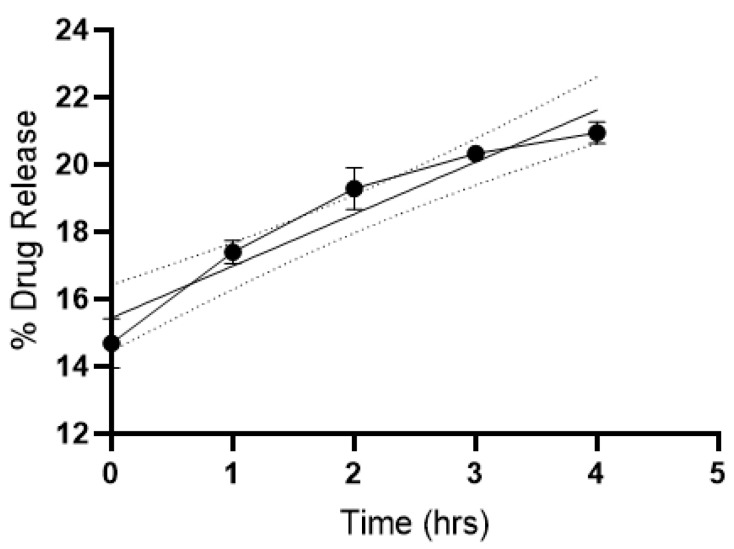
Release of TMZ in vitro from SLNP-TMZ at different time intervals (0–4 h) at pH = 7.0. The experiment was conducted in triplicate and the dotted lines represent the 95% confidence interval for the mean values, the data were plotted as mean ± s.e.m.

The drug release efficiency of the SLNPs was estimated over 0, 1, 2, 3, and 4 h time intervals post-suspension in PBS (pH = 7). The direct relation between time (h) and drug release was observed (Figure 8), indicating a slow and steady release of TMZ from the SLNPs.

### 3.3. Development of TMZ-Resistant U87-R Cell Line

For the generation of the temozolomide-resistant GBM cell lines, U87-MG, cells were subjected to periodic exposure of temozolomide, with a drug-free period of 72 h. Each time, the dose was moved to a higher concentration, up to 960 µmol/L. The treatment regimen was repeated for eight weeks to make sure that only resistant cells outgrew and gave rise to resistant cell lines. Before each passage, 90% cell confluency was achieved.

**Figure 9:**
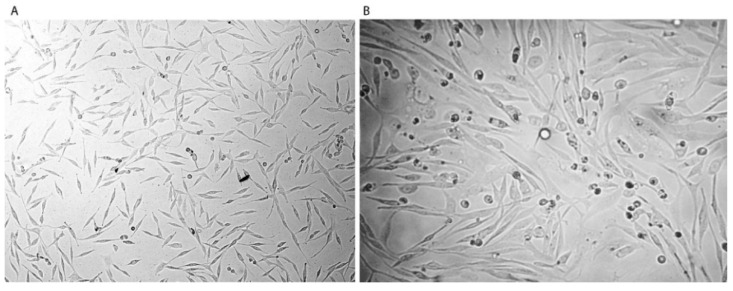
Morphology of U87-MG cells (U87-S) (100X magnification); (**A**) Before (**B**) After treatment with periodic exposure to temozolomide produces a chemo-resistant phenotype with longer extrusions and irregular shapes.

A cytotoxicity assay was performed with a sensitive U87-S cell line as a reference. Morphological changes were observed in the chemo-resistant phenotype (Figure 9A,B).

#### 3.3.1. Confirmation of Establishment of Resistance in U87-R

**Figure 10:**
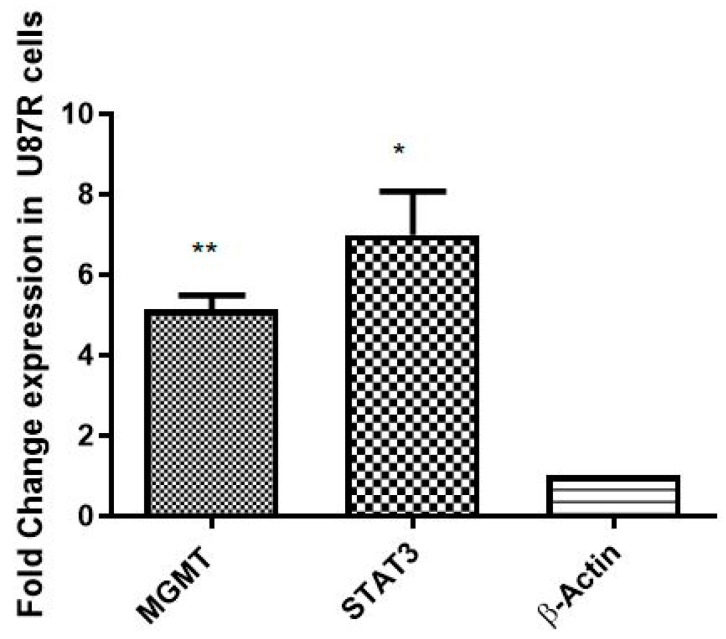
Fold change in expression of MGMT and STAT3 (resistance markers) in U87-S and U87-R. Each bar shows the mean ± SD values for each gene; experiments were conducted in triplicate. The results are normalized to β-actin, a housekeeping gene, and are expressed as a change from their respective controls. The level of significance is ** *p* ≤ 0.01, * *p* ≤ 0.05.

The establishment of a resistant cell line (U87-R) from TMZ-sensitive U87-S was confirmed by evaluating the expression of MGMT and STAT3 markers [25,26]. Significant elevation in MGMT (*p* = 0.0068) and STAT3 (*p* = 0.0307) mRNA expression, quantified by qRT PCR, confirmed the resistant nature of the U87-R cell line (Figure 10).

#### 3.3.2. Assessment of Proliferation and Anti-Apoptotic Markers in U87-R

**Figure 11:**
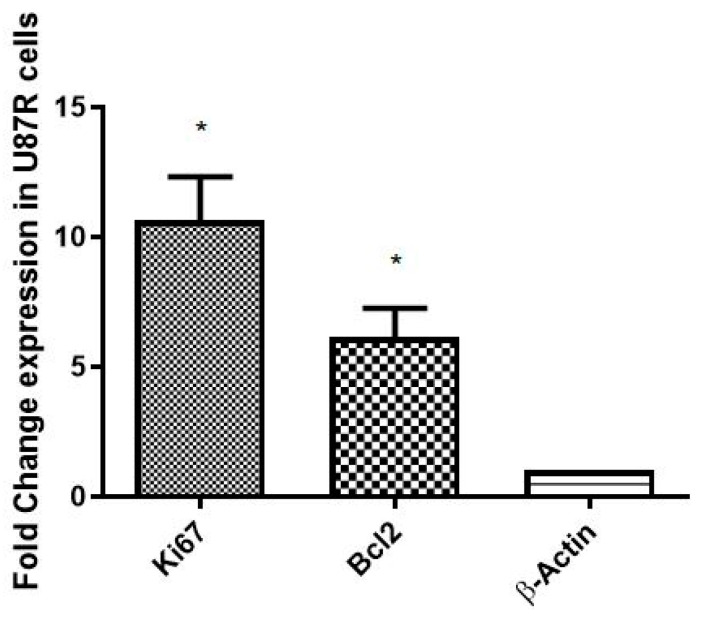
Fold change in expression of Ki67 (proliferation marker) and bcl2 (anti-apoptotic marker) in U87-S and U87-R. Each bar shows mean ± SD values for each gene; experiments were conducted in triplicate. The results are normalized to β-actin, a housekeeping gene and are expressed as a change from their respective controls. The level of significance is * *p* ≤ 0.05.

The rate of proliferation of resistant tumor cells (U87-R) was compared with the respective sensitive cell line (U87-S) via the level of Ki-67 (proliferation signatory marker). The level of the anti-apoptotic marker, Bcl2, was also quantified to evaluate the rate of apoptosis in U87-R cells. A significant elevation in the mRNA expression of Bcl2 (*p* = 0.0455) and ki67 (*p* = 0.0298) in U87-R tumor cells, as quantified by qRT PCR, was observed (Figure 11).

### 3.4. Effect of TMZ and SLNP-TMZ on the Proliferation of U87-S and U87-R Cells

**Figure 12:**
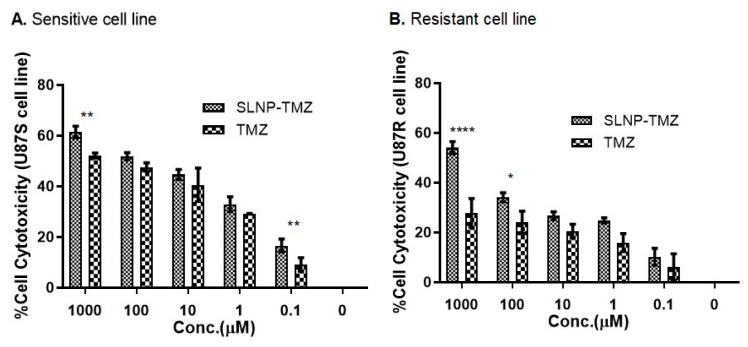
The cytotoxicity assay of TMZ and SLNP-TMZ (0.1–1000  µM) in U87-S (**A**) and U87-R (**B**) following an incubation period of 24 h. Experiments were repeated three times independently, and each assay was performed in triplicate. * *p* ≤ 0.05, ** *p* ≤ 0.01, **** *p* ≤ 0.0001.

The cytotoxicity of TMZ and TMZ -SLNPs was assessed on sensitive and resistant GBM cell lines by MTT assay at various concentrations from 0.1 µM to 1000 µM of TMZ and SLNP-TMZ formulations. Our results indicate that the SLNP-coated drug, SLNP-TMZ showed better anti-cancer potential in the case of both sensitive (Figure 12A) and resistant cell lines (Figure 12B) in a dose-dependent manner compared to naked TMZ.

### 3.5. Cell Migration by Wound Healing Assay

**Figure 13:**
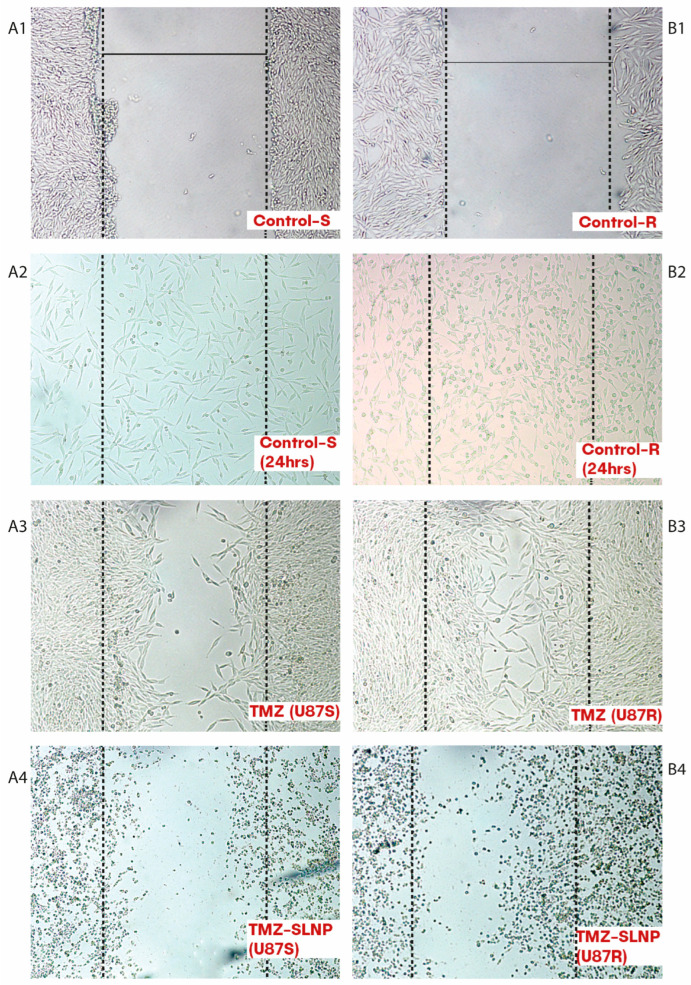
Cell migration assay (wound healing assay) Micrographs represent the effect of free TMZ and SLNP-TMZ on U87-S and U87-R cell lines after 24 h. All the images were taken at 100X magnification. (**A1**,**B1**) Represent the scratch made by using a 200 uL sterilized tip. (**A2**,**B2**) Represent the proliferation of cells without any treatment. (**A3**,**B3**) Show the migration of cells in the presence of free TMZ drug, the proliferation rate of the resistant cell line was greater than the sensitive one. (**A4**,**B4**), Shows the effect of SLNP-TMA treatment; a decrease in migration rate was observed along with cell death.

A cell migration assay was performed to determine the effect of SLNP-TMZ on the migratory/metastatic potential of sensitive and resistant cells for 24 h post-treatment. Our results indicate that SLNP-coated TMZ reduced the cell migration and metastatic potential of the cancer cells in both sensitive (Panel A1–4) and resistant (Panel B1–4) cell lines (Figure 13).

### 3.6. Time-Dependent Drug Distribution/Pharmacokinetics Studies in Mice

**Figure 14:**
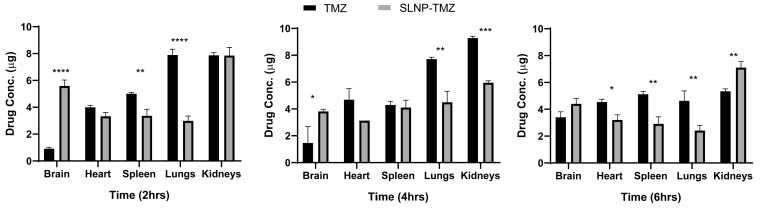
Temozolomide concentration in tissues at different time points (2 h, 4 h, 6 h) after intraperitoneal (i.p.) administration of TMZ and SLNP-TMZ (*n* = 3). The biodistribution profiles of temozolomide in mice are shown. Briefly, the drug concentrations in the brain were elevated after the administration of SLNP-TMZ compared to the temozolomide solution. Meanwhile, the drug concentration in the liver, kidney, heart, and spleen decreased in the case of SLNP-TMZ compared to the temozolomide solution. * *p* ≤ 0.05, ** *p* ≤ 0.01, *** *p* ≤ 0.001, **** *p* ≤ 0.0001.

The biodistribution profiles of temozolomide in mice after intraperitoneal administration of free TMZ and SLNP-TMZ were studied. Organs were harvested at 2 h, 4 h, and 6 h post-treatment with both formulations (TMZ and SLNP-TMZ). The amount of the naked TMZ (for naked TMZ and SLNP-TMZ-treated groups) in various organs of the mouse groups was determined using a spectrophotometer. Higher levels of the drug were observed in the heart, spleen, and lungs than in the brain when the drug TMZ was administered naked. However, a significant diversion of the drug from all other organs, except the spleen, towards the brain was observed when SLNP-coated TMZ was used. A significant shift from the spleen towards the brain was observed after 6 h. However, for all other organs, there was a reduction of TMZ accumulation when the drug was coated with SLNP, except for kidneys, in the later hours (Figure 14).

### 3.7. In Vivo Anti-Tumor Activity of SLNP-TMZ

**Figure 15:**
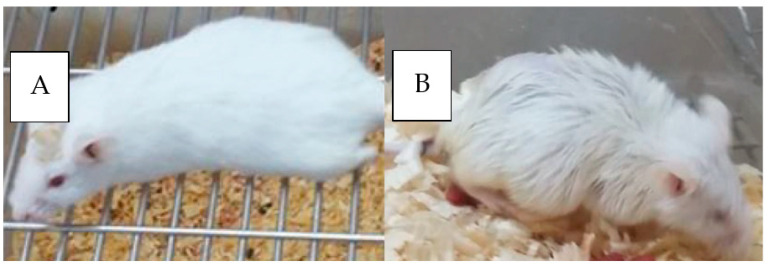
(**A**) Healthy mouse (**B**) Cancerous mouse.

The orthotopic xenograft mice models were generated and confirmed by Hematoxylin and Eosin staining. The induction of a tumor is evident by impaired mobility (the inability to reach food and water), hunched abnormal posture for more than 48 h, inability to remain upright, and weight loss in experimental tumor mice (Figure 15) [27].

**Figure 16:**
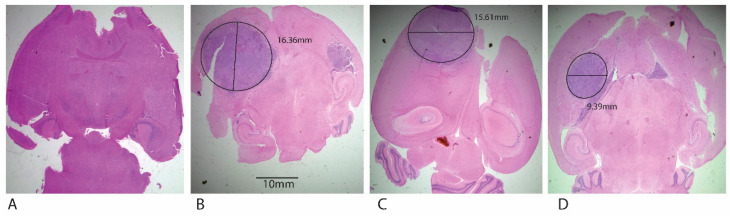
Comparison of the hematoxylin and eosin (H&E) staining in XM-S mice model after drug treatment (**A**) Negative control with no tumor, (**B**) Positive control after no drug treatment, (**C**) Tumor size after treatment with TMZ solution. (**D**) Tumor size after treatment with SLNP-TMZ. All the photographs were produced using light microscopy at 4X magnification.

After 7 days of tumor induction, XM-S groups were treated with TMZ and SLNPs-TMZ while control mice were administered with PBS solution only. The results showed a significant decrease in tumor size post-treatment, as depicted in HE stains of whole brain sections. No tumor was present in the negative control mice. A large size tumor of width 15.61 mm was seen in a TMZ solution-treated tumor, whereas SLNP-TMZ treatment considerably reduced the tumor width to 9.39 mm (Figure 16). All the images were taken at 4X magnification with the light microscope, and the tumor width was measured by Image J software.

### 3.8. In Vitro and In Vivo Expression of Signatory Inflammation Markers (IL-6, IL-1β, and TNF-α) GBM

**Figure 17:**
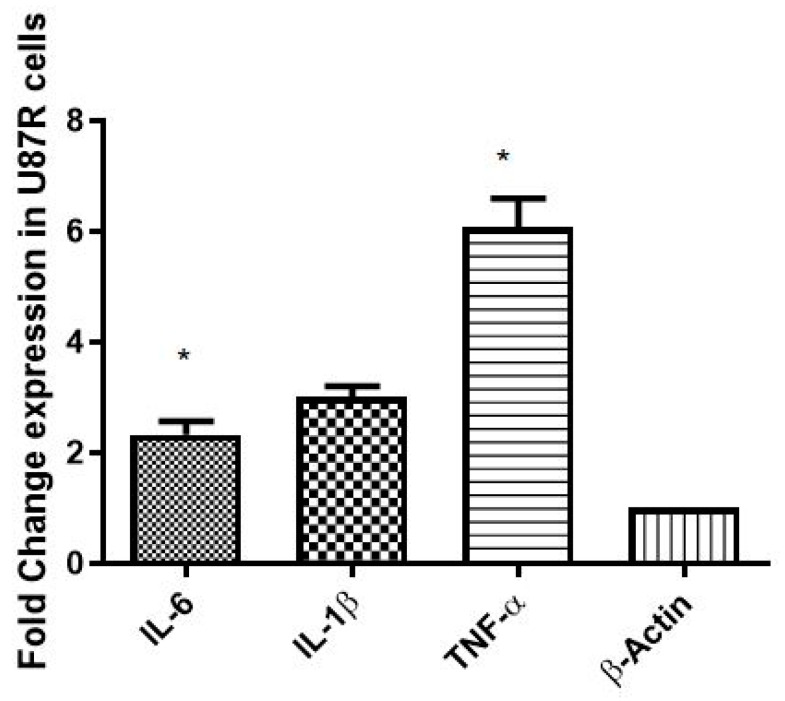
Fold change in gene expression of IL-6, IL-1β, and TNF-α (inflammatory markers) in U87-S and U87-R cell lines in an in vitro analysis. Each bar shows mean ± SD values for each gene. Experiments were conducted in triplicate. The results are normalized to β-actin, a housekeeping gene, expressed as a change from their respective controls. The level of significance is * *p* ≤ 0.05.

**Figure 18:**
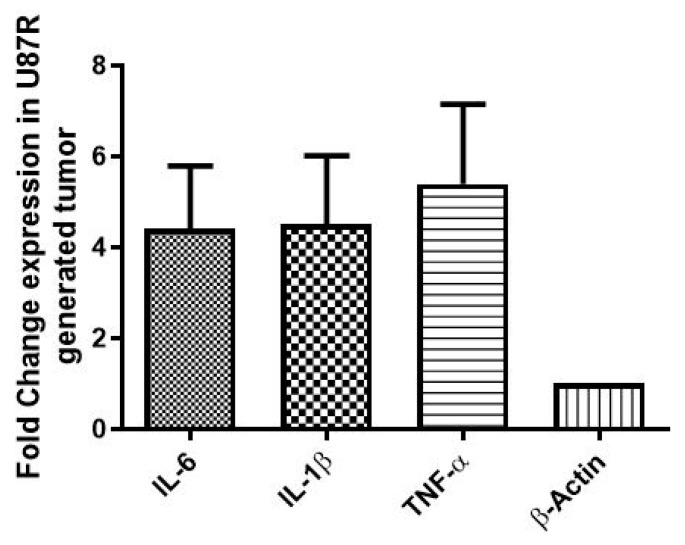
Fold change in gene expression of IL-6, IL-1β, and TNF-α (inflammatory markers) in XM-S and XM-R mice in an in vivo analysis. Each bar shows mean ± SD values for each gene. Experiments were conducted in triplicates. The results are normalized to β-actin, a housekeeping gene, expressed as a change from their respective controls.

The expression of inflammatory markers (IL-6, IL-1β, and TNF-α) with acquired drug (TMZ) resistance (both in vitro and in vivo) was evaluated by qRT-PCR. The results indicate the elevated expression of all inflammatory markers in the U87-R cell line compared to the U87-S cell line (*p* ≤ 0.05) (Figure 17). Similar results were obtained in vivo where the elevated expression of IL-6 (approximately twofold), IL-1β (threefold), and TNF-(sixfold) was observed (the expression of the stated inflammatory markers was evaluated in XM-S and XM-R mouse models after 21 days of tumor induction(Figure 18)). Interestingly, the levels of these markers in vivo were elevated by nearly double that of the in vitro models (Figure 18).

## 4. Discussion

Temozolomide is currently the most effective drug against glioma; it is considered standard chemotherapy in combination with radiotherapy after surgery [28]. Despite its efficient activity against GBM tumors, it has certain limitations, such as at the required dose, it causes systemic toxicity, as do most chemotherapies. The blood-brain barrier (BBB), being the brain shield, causes hindrance in the delivery of drugs to the target area within the brain [29]. The recurrence of tumors and acquisition of resistance by GBM tumor cells is yet an unsolved problem [30].

The challenges presented by the BBB and the systemic toxicity of temozolomide could be overcome by nanocarrier systems. Nano-sized solid lipid nanoparticles (SLNPs) are formulated in sizes ranging between 20 nm and 200 nm and can cross the barriers of the brain. These solid lipid nanoparticles (SLNPs) are composed of stearic acid and lecithin, coated with polysorbate-80 (PS-80). Therefore, no toxic effects can be expected from these base molecules upon degradation. The lipid nature of SLNPs allows their passage through the cell membrane, and the dense network of capillaries around the brain allows its passage through the BBB [22].

Therefore, the enhanced anti-cancer potential of SLNPs conjugated TMZ was accessed in-vitro and in-vivo using U87-S and U87-R cell lines and a rodent model of a GBM tumor, respectively. Resistant cell lines were generated from the sensitive U87-S cell line and confirmed by *Ki67* and *bcl-2*, proliferation, and anti-apoptotic markers in the U87-R cell line [31,32].

Characterization of SLNPs showed their potential to be used as delivery vehicles. Scanning electron micrographs showed the spherical morphology of the SLNPs [33]. To determine the degree of crystallinity of the drug incorporated in SLNPs, XRD analysis showed sharp peaks of TMZ at angles of 26 and 28 degrees; these peaks became less intensified in conjunction with the presence of SLNPs, which shows lower crystallinity of the drug, and this can benefit the incorporation with SLNPs. Also, the degradation of SLNPs is easier at a lower crystallinity [34,35]. FTIR analysis showed that most of the same functional groups are present in the drug and drug-conjugated SLNPs; this indicates the presence of the drug in formulated SLNPs. The presence of the PS-80 coating was also confirmed by FTIR analysis. The entrapment efficiency of SLNPs came to be 94.47%; this shows the efficient ability of SLNPs to entrap TMZ. The in vitro time-dependent release of TMZ was also monitored, and sustained release was observed.

The decrease in cancer cell viability with SLNP-TMZ in resistant cell lines also presents the striking potential of SLNPs in managing the resistant nature of tumor cells. A similar trend was observed in one of the published studies, in which free doxorubicin and dox-nanoparticles were used to treat resistant Hela cells. The MTT results showed drug efficiency enhancement in sensitive and resistant Hela cell lines due to increased drug retention time in the cytosol and sustained drug release, which increased the timeframe of activity of the drug without losing its potential [36].

In vitro cytotoxicity analysis was performed on the GBM cell lines. The effect of SLNPs conjugated drug was evaluated on two types of GBM cell lines; one was TMZ-sensitive, and the other was a parental-derived, TMZ-resistant cell line (U87-R). The percentage of cell viability decreased when SLNP-TMZ was administered to the cell culture compared to the TMZ-solution at varying concentrations; the same trend was followed in both types of cell lines, but more cell killing was seen in the TMZ-sensitive cell line than the resistant cells with SLNP-TMZ. Nevertheless, SLNPs were seen to be more effective in the resistant cells than the naked drug. A similar trend was seen in a related study where the anti-cancer activity of Paclitaxel-incorporated solid lipid nanoparticles (Ptx-SLNs) was checked against the drug-sensitive breast cancer line MCF7 and its multi-drug resistant variant MCF/ADR. Remarkably, enhanced anti-cancer activity was found in MCF7/ADR with Ptx-SLNs rather than free Ptx due to the significant increase in intracellular uptake of Ptx with SLNs [37]. These results are in line with our current study.

For in vivo testing, SLNP-TMZ was administered to GBM orthotopic xenograft mouse models. H&E staining showed a reduction of tumor size in the case of SLNP-TMZ. Conversely, large-size tumors (9.39 mm) were found in the animal group treated with free TMZ, with a dense population of tumor cells characterized by high nuclei distribution. In contrast, a smaller tumor size (5.04 mm) was observed in the group administered with SLNP-TMZ. A similar trend was present in a study related to breast cancer where curcumin-loaded solid lipid nanoparticles (CURC-SLNs) were tested against doxorubicin-resistant triple-negative breast cancer cells. The results showed that CURC-SLNs were five- to tenfold more effective than free CURC, with an increase in intracellular retention of the drug and no signs of systemic toxicity [38].

The tissue distribution of SLNP-TMZ after administration in mouse models showed significantly higher concentrations of the drug in the brain than in TMZ alone. The targeted delivery of the drug to the brain is attributed to the blood apolipoprotein E that gets adsorbed onto the surface of the SLNPs due to the PS-80 coating, which mimics the LDL proteins that are recognized by LDL receptors present on the BBB’s endothelial cells [39]. It opens the tight junctions and increases the permeation of nanoparticle-bound drug that gets internalized by the mechanism of receptor-mediated endocytosis [40].

The tissue distribution results demonstrated that the SLNP formulation for drug delivery was appropriate for GBM, as a greater amount of the drug was transported into the brain compared to the free TMZ; direct release of the drug into the brain is beneficial for the treatment of tumors. The total sum of the drug in different organs was less when administered with SLNPs than the free drug, which is important because it would reduce systemic toxicity such as nephric or cardiac toxicity. One of our previously published studies of diosgenin incorporated solid lipid nanoparticles (Dio. SLNP), which also showed matching results, where a significant increase of diosgenin in the brain was found with SLNPs, with a reduction of drug concentration in other vital organs [22]. In another study in which SLN-DiR and Free-DiR were used, the same trend in the context of tissue distribution was observed, which was in line with our present study [41].

After finding the significant anti-cancer effect of SLNP-coated TMZ both in vitro and in vivo, the potential resistance mechanism of GBM cells against TMZ was evaluated. Important inflammatory markers, including IL-6, IL-1β, and TNF-α, were studied to elucidate their role in the resistance of GBM cells against temozolomide.

The purpose of this study was to inspect the role of these downstream genes in TMZ-resistant GBM cells, to validate whether inflammation is related to resistance or not and to have a better understanding of the players of GBM resistance. The proliferation and anti-apoptotic potential of tumor cells are also attributed to the elevated levels of IL-6 [42], IL-1b [43], and TNF-α [44]. Therefore, levels of expression of these inflammatory cytokines were assessed in TMZ-resistant cells.

Expression of inflammatory markers (IL-6, IL-1β, and TNF-α) was found to be elevated in TMZ-resistant cells compared to the sensitive cells in both cell culture models and mouse models. These results are in line with previous studies, where the role of IL-6 in gastric cancer was studied, and it was observed that in chemo-resistant cancer-associated fibroblast cells, the levels of IL-6 and the downstream signaling molecule STAT-3 were higher than in the normal fibroblast cells [45]. One of the related studies of IL-1β is with acute lymphoblastic leukemia, in which IL-1β is known to induce NF-κB activation; more importantly, in KRAS mutant cancer cells, it leads to drug resistance and increased cancer progression [46]. Similarly, the expression of cytokines TNF-α, IL-1, and IL-6 was found to be increased in drug-resistant colorectal cancer in the blood samples of each group of xenograft mouse models [47].

Our study demonstrated that TMZ, in conjunction with SLNPs, induces notable anti-tumor effects and presents less toxicity in the in-vivo models. More importantly, it can pass through the BBB in vivo, so it provides an efficient means to deliver drugs into the brain to treat different brain abnormalities. The role of IL-6, IL-1β, and TNF-α, along with MGMT and STAT-3, in the resistance mechanism of GBM against TMZ is evident from our findings, and the effect of delivery vehicles on minimizing the inflammatory environment is also evident from the current study.

Solid lipid nanoparticles can be applied in the various treatment approaches for cancers and other systemic abnormalities. Inflammatory markers can be targeted to develop anti-inflammatory drugs to be administered concomitant with TMZ to produce a synergistic effect. This could lower the expression of inflammatory resistant signatory molecules and attenuate GBM resistance against TMZ, thus opening avenues for an effective strategy against the acquired resistance and recurrence phenomena of GBM tumors.

## 5. Conclusions

A preliminary study suggests SLNPs as a better carrier of TMZ for effective and targeted drug delivery to the brain. The role of IL-6, IL-1β, and TNF-α, along with MGMT and STAT-3, in the resistance mechanism of GBM against TMZ is evident. Nano-coated drugs were found to reduce the tumor size in chemotherapy-sensitive and resistant cells significantly. The SLNPs assist in the transport and release of the loaded therapeutic to the target site, which reduces the off-target accumulation of the payload and leads to reduced cytotoxicity that effectively fights drug resistance in cancer cells.

The prospects of further study include the following: as the concentrations used for treating the GBM in these experiments did not cause a complete loss of the tumor, analysis to check whether the remaining cells can re-grow back into the tumor is needed to grade the potential of this treatment for treating GBM. Moreover, only the effect of TMZ and their induced inflammatory factors in vitro and in vivo were detected, indicating that drug resistance was related to inflammation. However, the dose-dependent effect of TMZ and SLNP-TMZ on inflammatory markers is yet to be analyzed, requiring our further experimentation.

## Figures and Tables

**Table 1:** Experimental groups for in vivo analysis of the cytotoxic potential of SLNP-TMZ and free TMZ.

Sr No.	Groups	Treatment	Duration
1	PBS (Control)	TMZ	21 Days (5 times a week)
2	PBS (Control)	SLNP-TMZ	21 Days
3	Sensitive-cell-induced tumor	PBS	21 Days
4	Sensitive-cell-induced tumor	TMZ	21 Days
5	Sensitive-cell-induced tumor	SLNP-TMZ	21 Days

**Table 2:** Primer Sequences designed for qPCR.

Sr No.	Genes	Primers
1	β-actin (*Homo sapiens*)	F:CATGTACGTTGCTATCCAGGC R:CTCCTTAATGTCACGCACGAT
2	MGMT (*Homo sapiens*)	F:TTTTCCAGCAAGAGTCGTTCAC R:GGGACAGGATTGCCTCTCAT
3	STAT-3 (*Homo sapiens*)	F:ACCAGCAGTATAGCCGCTTC R:GCCACAATCCGGGCAATCT
4	Ki67 (*Homo sapiens*)	F:ATCATTGACCGCTCCTTTAGGT R:GCTCGCCTTGATGGTTCCT
5	Bcl-2 (*Homo sapiens*)	F:CATGTGTGTGGAGAGCGTCAA R:GCCGGTTCAGGTACTCAGTCA
6	IL-1β (*Homo sapiens*)	F:ACGATGCACCTGTACGATCA R:TCTTTCAACACGCAGGACAG
7	IL-6 (*Homo sapiens*)	F:AGGAGACTTGCCTGGTGAA R:CAGGGGTGGTTATTGCATCT
8	TNF-α (*Homo sapiens*)	F:TGGAGAAGGGTGACCGACTC R:TGCCCAGACTCGGCAAAG
9	β-actin *(Mus musculus*)	F:GGCTGTATTCCCCTCCATCG R:CCAGTTGGTAACAATGCCATGT
10	IL-1β *(Mus musculus*)	F:CAGGCAGGCAGTATCACTCA R:AGCTCATATGGGTCCGACAG
11	IL-6 *(Mus musculus*)	F:AGTTGCCTTCTTGGGACTGA R:TCCACGATTTCCCAGAGAAC
12	TNF-α *(Mus musculus*)	F:CAGGCGGTGCCTATGTCTC R:CGATCACCCCGAAGTTCAGTAG

**Table 3:** Elemental composition of SLNPs through Energy Dispersive Spectroscopy (EDS).

Compound	Element	Weight%	Atomic%
SLNP-TMZ	C	25.91	33.47
	O	61.83	59.96
Blank-SLNPs	C	26.54	35.49
	O	50.37	50.56

## Data Availability

The raw data supporting the conclusions of this article will be made available by the authors without undue reservation.
